# Inhibitory Peptide of Mitochondrial μ-Calpain Protects against Photoreceptor Degeneration in Rhodopsin Transgenic S334ter and P23H Rats

**DOI:** 10.1371/journal.pone.0071650

**Published:** 2013-08-09

**Authors:** Taku Ozaki, Sei-ichi Ishiguro, Satoshi Hirano, Ayaka Baba, Tetsuro Yamashita, Hiroshi Tomita, Mitsuru Nakazawa

**Affiliations:** 1 Department of Ophthalmology, Hirosaki University Graduate School of Medicine, Hirosaki, Japan; 2 Department of Biochemistry and Molecular Biology, Hirosaki University Faculty of Agriculture and Life Science, Hirosaki, Japan; 3 Department of Biological Chemistry, Iwate University Faculty of Agriculture, Morioka, Japan; 4 Department of Chemistry and Bioengineering, Iwate University Graduate School of Engineering, Morioka, Japan; Massachusetts Eye & Ear Infirmary, Harvard Medical School, United States of America

## Abstract

Mitochondrial μ-calpain and apoptosis-inducing factor (AIF)-dependent photoreceptor cell death has been seen in several rat and mouse models of retinitis pigmentosa (RP). Previously, we demonstrated that the specific peptide inhibitor of mitochondrial μ-calpain, Tat-µCL, protected against retinal degeneration following intravitreal injection or topical eye-drop application in *Mertk* gene-mutated Royal College of Surgeons rats, one of the animal models of RP. Because of the high rate of rhodopsin mutations in RP patients, the present study was performed to confirm the protective effects of Tat-µCL against retinal degeneration in rhodopsin transgenic S334ter and P23H rats. We examined the effects of intravitreal injection or topical application of the peptide on retinal degeneration in S334ter and P23H rats by terminal deoxynucleotidyl transferase-mediated dUTP nick-end labeling (TUNEL) assay, electroretinogram (ERG), immunohistochemistry for AIF, and histological staining. In S334ter rats, we found that intravitreal injection or topical application of the peptide prevented photoreceptor cell death from postnatal (PN) 15 to 18 days, the time of early-stage retinal degeneration. Topical application of the peptide also delayed attenuation of ERG responses from PN 28 to 56 days. In P23H rats, topical application of the peptide protected against photoreceptor cell death and nuclear translocation of AIF on PN 30, 40, and 50 days, as the primary stages of degeneration. We observed that topical application of the peptide inhibited the thinning of the outer nuclear layer and delayed ERG attenuations from PN 30 to 90 days. Our results demonstrate that the mitochondrial μ-calpain and AIF pathway is involved in early-stage retinal degeneration in rhodopsin transgenic S334ter and P23H rats, and inhibition of this pathway shows curative potential for rhodopsin mutation-caused RP.

## Introduction

Retinitis pigmentosa (RP) is a hereditary retinal degeneration characterized by night blindness, photophobia, gradual loss of the peripheral visual field, color blindness, and eventual visual disturbance. These symptoms are caused by progressive rod photoreceptor degeneration in the early stage, followed by eventual cone photoreceptor degeneration. The disease prevalence is about 1/4,000–5,000, and the condition is common around the world. The hereditary characteristics are heterogeneous, and characterized by autosomal-dominant (ADRP), autosomal-recessive (ARRP) or X-linked inheritance patterns. Recent molecular genetic studies have also revealed that more than 100 different genes are involved in or cause RP (Ret-Net: http://www.sph.uth.tmc.edu/retnet/disease.htm).

Despite the numerous gene mutations, RP occurs in association with rod photoreceptor apoptosis as a common pathway [1]. This apoptosis has been detected in animal models of RP such as retinal degeneration 1 (rd1), retinal degeneration slow (rds), and rhodopsin (Rho) mutant mice [2]. Photoreceptor cell death is also known to be caused by many pathways involving caspases, cathepsins, calpains, apoptosis-inducing factor (AIF), oxidative stress, endoplasmic reticulum (ER) stress, poly(adenosine diphosphate-ribose) polymerase (PARP), etc. [1], [3]–[5]. However, recent studies have revealed that calpains and/or AIF cause photoreceptor cell death in Royal College of Surgeons (RCS), Rho S334ter, and Rho P23H rats, and rd1, rd10, and Rho T17****M mice [3], [4], [6]–[10]. These results are supported by many reports showing that intracellular concentrations of calcium ions are elevated during photoreceptor degeneration in the rat and mouse models of RP [1].

Our previous studies demonstrated that calcium ions, calpain, and AIF are the main causes of photoreceptor cell death in RCS rats in the early stages of retinal degeneration [1], [6], [7], [11]. First, Yamazaki *et al* found that a low-voltage-activated calcium channel blocker, nilvadipine, preserves retinal morphology and functions in RCS rats [11]. Those results suggested that intracellular concentrations of calcium ions are elevated, and calpains, as calcium-dependent cysteine proteases, are activated in the photoreceptor. Second, we showed that mitochondrial calpain is activated and truncates AIF, followed by the release of truncated AIF (tAIF) from the mitochondria into the nucleus in the initial stage of retinal degeneration in RCS rats [6]. It is well known that after truncation of AIF by mitochondrial μ-calpain [12]–[20], tAIF can translocate from the mitochondrial inner membrane to the nucleus, where it facilitates chromatin condensation and large-scale DNA fragmentation [21], [22]. We also found that intravitreal injection of the calpain inhibitors ALLN and PD150606 at the time of mitochondrial calpain activation transiently inhibited nuclear translocation of tAIF and photoreceptor apoptosis [6]. Inhibition of the mitochondrial μ-calpain-AIF pathway would thus provide significant benefit in the treatment of RP.

Recently, we found that a specific peptide inhibitor of mitochondrial μ-calpain, Tat-µCL (another name for HIV-Nμ), transiently prevents retinal degeneration and attenuation of electroretinogram (ERG) response following intravitreal injection or eye-drop application in RCS rats [7]. The RCS rat carries a mutation in the *Mertk* gene expressed in the retinal pigment epithelium (RPE), and this mutation has been characterized in ARRP [23]. However, because the mutation is only one of many gene mutations causing RP, we still do not know whether the results from that previous study [7] can be generalized to other types of RP associated with defects genes other than the *Mertk* gene, or are instead specific to RP caused by mutations in the *Mertk* gene. To obtain clues for solving this question, we need to examine the effects of Tat-µCL on RP models other than the RCS rat. Because RP is genetically highly heterogeneous, molecular mechanisms that lead to photoreceptor apoptosis may also differ according to the causative genes. The present study, therefore, examined the protective effects of Tat-µCL against retinal degeneration using other RP models, namely Rho transgenic S334ter and P23H rats, as well-known models for ADRP [24]–[26].

Calpains and/or AIF play a significant role in the photoreceptor degeneration of both S334ter and P23H rats [3], [4]. Shinde *et al* demonstrated that calpains are activated and AIF is released from the mitochondria to the cytosol in the initial stage of photoreceptor cell death in S334ter rats [4]. Furthermore, Kaur *et al* reported that the calpain-dependent pathway, but not the caspase-dependent pathway, contributes to photoreceptor cell death in P23H rats [3].

Accordingly, the purpose of the present study was to determine whether the mitochondrial μ-calpain inhibitory peptide, Tat-µCL, protects against retinal degeneration in both S334ter and P23H rats. Because degeneration progresses more rapidly in S334ter rats than in P23H rats, we examined the short-term protective effects of Tat-µCL against photoreceptor cell death and function in S334ter rats, and long-term protective effects in P23H rats.

## Materials and Methods

### Animals

All experimental procedures were designed to conform to the Association for Research in Vision and Ophthalmology (ARVO) Statement for Use of Animals in Ophthalmic Vision Research and were approved by the Committee for the Use of Live Animals (Permit Number: M10016) at the Hirosaki University. Sprague-Dawley (SD) rats were purchased from Clea Japan (Tokyo, Japan), and used as wild-type (wt) controls. Homozygous S334ter (line 4) and P23H (line 2) Rho transgenic rats were generously provided by Dr. Matthew M. LaVail (University of California), and were housed at the Hirosaki University Graduate School of Medicine Animal Care Service Facility under a 12-h light (50 lux illumination) and 12-h dark (<10 lux illumination) cycle. Care was taken not to cause photoreceptor light damage to rats.

### Synthesis of μ-calpain C2L Domain Peptides

We separately synthesized Tat-µCL (GRKKRRQRRRPPQ-PDALKSRTLR, 23 aa; molecular weight (MW), 2857.37 Da) and its scramble peptide (GRKKRRQRRRPPQ- ASLRLDRPTK, 23 aa; MW 2857.37 Da), as described in our previous study [7]. Each peptide was synthesized by the fluorenylmethyloxycarbonyl method using an automated peptide synthesizer (Shimazdu PSSM-8; Shimazdu, Kyoto, Japan). The resulting peptides were purified by reverse-phase HPLC using a C18 column (Jupiter 250 mm×10 mm; Phenomenex, Torrance, CA). The molecular weight and purity of each peptide was confirmed by MALDI-TOF mass spectrometry with a Voyager RP-DE (Applied Biosystems, Foster City, CA). Purity of each synthesized peptide was >95% as estimated from the relative absorbance by HPLC.

### Subcellular Fractionation of Rat Retinas

Subcellular fractionation of S334ter or P23H rat retinas was performed as described [6], [27]. All experimental procedures were carried out at 4°C. Rats were sacrificed with inhalation of carbon dioxide. After enucleation, eyes were washed in ice-cold phosphate-buffered saline (PBS) (0.14 M NaCl and 10 mM phosphate buffer, pH 7.4) and dissected into halves. Retinas taken from both eyes of each rat were homogenized in 500 µl of homogenizing buffer (20 mM Tris-HCl, pH 7.5, containing 1 mM ethylene diamine tetraacetic acid (EDTA), 1****mM ethylene glycol tetraacetic acid (EGTA), 0.25 M sucrose and 5 mM 2-mercaptoethanol) with a 2-ml-glass-teflon homogenizer. The homogenate was centrifuged at 600×g for 5 min to remove the nuclear fraction, and the supernatant was then centrifuged at 4,500×g for 10 min to obtain the mitochondrial fraction. The supernatant was then centrifuged at 20,000×g for 20 min to remove the lysosomal fraction. The supernatant was used as a cytosolic fraction. The mitochondrial fraction was suspended in 200 µl of suspending buffer (20 mM Tris-HCl, pH 7.5, containing 1 mM EDTA, 1 mM EGTA, and 5 mM 2-mercaptoethanol), and sonicated to disrupt the mitochondrial inner and outer membrane.

### Western Blot Analysis

Western blot analyses were performed to detect and determine the quantity of AIF, as previously described [28], [29]. Mitochondrial or cytosolic proteins of S334ter or P23H rat retinas were subjected to sodium dodecyl sulfate (SDS)-polyacrylamide gel electrophoresis (PAGE) on 10% SDS-polyacrylamide gels (20–40 µg proteins per lane). Western blotting was performed after SDS-PAGE. Immunoreactive signals were developed with an enhanced chemiluminescence Western blotting detection kit (Amersham Biosciences, Buckinghamshire, UK) and quantified with a luminescent image analyzer (LAS-3000; Fujifilm Co., Tokyo, Japan).

### Administration of Tat-µCL

We determined the short- and long-term effects of Tat-µCL using S334ter and P23H rats, respectively. For S334ter rats, we performed intravitreal injection of 20 mM Tat-µCL at postnatal (PN) 15 days or topical eye-drop application of 20 mM Tat-µCL twice a day on PN 13–17 days for terminal deoxynucleotidyl transferase-mediated dUTP nick-end labeling (TUNEL) and PN 13–55 days for ERG. For P23H rats, we performed topical eye-drop application of 1 mM Tat-µCL twice a day on PN 14–39 days for immunohistochemical study, PN 14–49 days for TUNEL assay, and PN 14–89 days for ERG and light microscopic studies. Assuming a volume of rat vitreous of 30 µl, the final concentration of peptide in the vitreous was considered to be 1.25 mM immediately after intravitreal injection. However, we estimated that the concentration of peptide in the mitochondria of photoreceptor cells would be within the range of one-tenth to one-hundredth of 1.25 mM, i.e, 12.5–125 µM, because the peptides would be diluted by ocular circulation, degraded by various proteases, and/or blocked by the several barriers such as extracellular matrices. These concentrations of 12.5–125 µM would be considerably higher than IC_50_ (197 nM) [7] and would exert no cytotoxic effects on the photoreceptors. In topical eye-drop application, we used 20 mM Tat-µCL for S334ter rats and 1 mM peptide for P23H rats, respectively. The concentration of the peptide was determined according to the severity of each RP model [30]. Because the P23H (line 2) has been demonstrated to be slowly progressive [30], we speculated that 1 mM Tat-µCL might be sufficient.

Rats were anesthetized by intramuscular injection of ketamine (80–125 mg/kg) and xylazine (9–12 mg/kg). After topical application of 0.02% oxybuprocaine hydrochloride to the cornea, each animal received an intravitreal injection of 2 µl of 20 mM Tat-µCL in PBS, 4 mM PD150606, or PBS (n = 6 per group). Solutions were injected using a Hamilton syringe with a 30-gauge needle through the area of the ciliary body (1 mm posterior to the corneal limbus) into the vitreous cavity. Special care was taken to avoid injury to the lens and the needle was penetrated vertically into the eyeball. Alternatively, topical eye-drop application was performed twice a day without anesthesia.

### TUNEL Assay

To determine retinal photoreceptor cell death, cleavage of DNA was visualized *in situ* by TUNEL assay as previously described [7]. The eyes of Tat-µCL-treated RCS rats were enucleated and embedded in OCT compound (Sakura, Tokyo, Japan). Cryosections (5 µm thick) were cut superior or inferior to the plane of the equator containing the optic disc and the central portion of the eyeball, respectively. Numbers of TUNEL-positive cells were counted in 20 sections per eye in nine eyes from each group. After washing with PBS, TUNEL was performed using *In Situ* Apoptosis Detection Kits (Takara, Ohtsu, Japan) according to the instructions from the manufacturer. Sections were counterstained with 4′-6′-diamidino-2-phenylindole (DAPI) to stain nuclei. Immunofluorescent images were acquired by laser scanning confocal microscopy (FV1000-D; Olympus, Tokyo, Japan).

### Immunohistochemistry

Immunohistochemistry to detect the localization of AIF was performed as previously described [6]. Enucleated eyes were fixed in 4% paraformaldehyde in PBS, pH 7.4, for 20 min at room temperature. Anterior segments were removed and posterior eyecups were placed in the same fixative overnight at 4°C. Eyecups were cryoprotected for 4 h in 10% then 20% sucrose in PBS, then frozen in OCT compound. Cryosections (5 µm thick) were made superior or inferior to the equator plane containing the optic disc and the central portion of the eyeball. Sections were rinsed in PBS, incubated with 0.3% hydrogen peroxide in methanol for 15 min, and blocked with 1% skim milk in PBS plus Tween (PBS-T) for 2 h at room temperature. Sections were incubated overnight at 4°C with the rabbit polyclonal anti-AIF antibody (ab1998, 1∶1000; Abcam, Cambridge, UK) diluted in 1% skim milk in PBS-T. Sections were then washed with PBS-T and incubated with horse radish peroxidase (HRP)-conjugated goat anti-rabbit immunoglobulin G (P0448, 1∶500; DAKO, Glostrup, Denmark) overnight at 4°C. Sections were washed with PBS-T and incubated with tetramethylrhodamine-labeled tyramide (PerkinElmer. Ins, Waltham, MA) for 10 min at room temperature. Sections were then washed with PBS-T and mounted with Vectashield (Vector, Burlingame, CA) with DAPI.

### ERG

The methods for measurement of ERG response were described in our previous study [7]. The time points for ERG measurement were determined according to the time courses of photoreceptor degeneration in S334ter (line 4) and P23H (line 2) rats [30]. ERGs were recorded at PN 18, 21, 28, 35, 42, 49 and 56 days for S334ter rats, and at PN 30, 70 and 90 days for P23H rats, respectively. Briefly, S334ter or P23H rats were moved to an electrically shielded room, anesthetized, and laid on their side on a heating pad (at 37°C). The head was fixed in place with surgical tape, and the rats were dark-adapted for 24 h. The pupils were dilated with 0.5 mg/ml tropicamide and 0.5 mg/ml phenylephrine hydrochloride eye-drops. ERGs were recorded with a contact electrode equipped with a suction apparatus to fit on the cornea (Kyoto Contact Lens, Kyoto, Japan). A grounding electrode was placed on the nose. Responses to a 200-ms duration white flash (3.5 ×10^2^ lux) were recorded (Neuropack, model MES-3102; Nihon Kohden, Tokyo, Japan). The a-wave amplitude was determined as the baseline to the bottom of the a-wave. The b-wave amplitude was determined as the bottom of the a-wave to the top of b-wave. ERG amplitudes are shown as mean ± standard deviation.

### Hematoxylin and Eosin Staining

Hematoxylin staining was performed with New Hematoxylin Type M solution (Muto Pure Chemicals, Tokyo, Japan) for 10 min. After washing under running water, eosin staining was performed with 1% eosin Y solution (Muto Pure Chemicals) for 30 sec. Sections were dehydrated with 70%, 80%, 90%, 95%, and 100% ethanol and xylene, then enclosed with MGK-S (Matsunami Glass, Osaka, Japan).

### Statistical Analysis

Student’s *t* test or analysis of variance (ANOVA) was used to statistically compare results. Experiments were performed in triplicate to confirm reproducibility.

## Results

### TUNEL Assay in S334ter Rat Retinas

**Figure 1:**
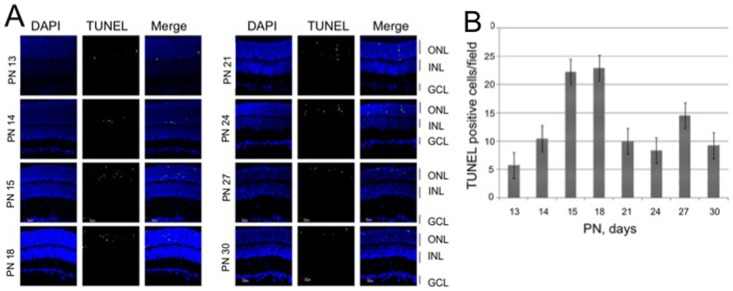
Determinations of photoreceptor cell death in S334ter rat retinas. **A)** TUNEL assay of retinal sections of S334ter rats. Eyes were enucleated at PN 13, 14, 15, 18, 21, 24, 27, or 30 days. Retinal sections were stained with TUNEL (green) and DAPI (blue). B) Quantitative analysis of the number of TUNEL-positive cells in the ONL. Data are expressed as means ± standard deviation (n = 12 eyes (6 rats) per group). Abbreviations: ONL, outer nuclear layer; INL, inner nuclear layer; GCL, ganglion cell layer.

To determine the time course of the photoreceptor degeneration in S334ter rat retina, we performed the TUNEL assay to detect the photoreceptor cell death (Figure 1). In the outer nuclear layer (ONL), the TUNEL-positive cells were detected from PN 13 days and reached the highest level at PN 15 and 18 days, while photoreceptors were still undergoing cell death at PN 21–30 days.

### Intravitreal Injection of Tat-µCL Prevents Photoreceptor Cell Death in S334ter Rats

**Figure 2:**
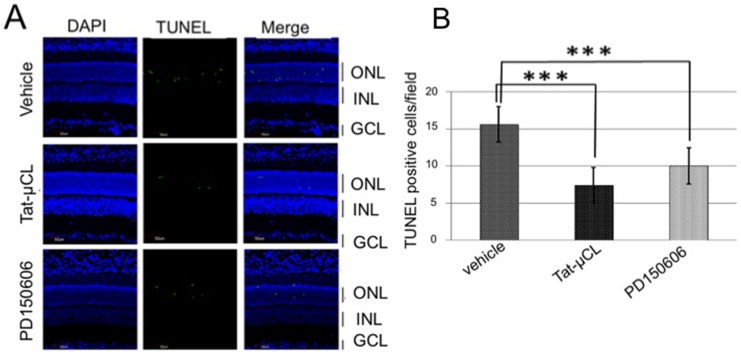
Effects of intravitreal injection of Tat-µCL on photoreceptor cell death in S334ter rats. A) TUNEL staining of retinal sections of S334ter rats treated with Tat-µCL. S334ter rats received intravitreal injection of 2 µl of vehicle (PBS), 4 mM PD150606, or 20 mM Tat-µCL at PN 15 days. Eyes were enucleated at PN 18 days. Retinal sections were stained with TUNEL (green) and DAPI (blue). B) Quantitative analysis of the number of TUNEL-positive cells in the ONL at PN 18 days. Data are expressed as means ± standard deviation (n = 12 eyes (6 rats) per group). ***P<0.001 versus vehicle (*t*-test). Abbreviations: ONL, outer nuclear layer; INL, inner nuclear layer; GCL, ganglion cell layer.

With intravitreal injection, we determined the protective effects of Tat-µCL against photoreceptor cell death in S334ter rats (Figure 2). Rats received an intravitreal injection of 2 µl of vehicle (PBS), 20 mM Tat-µCL, or 4 mM PD150606 (calpain inhibitor) at PN 15 days. Detection of TUNEL-positive cells was performed at PN 18 days. Results showed that the number of TUNEL-positive cells in the ONL was decreased with intravitreal injection of Tat-µCL (∼50% inhibition) and PD150606 (∼33% inhibition). Quantitative analysis of the number of TUNEL-positive cells showed that Tat-µCL or PD150606 significantly prevented the photoreceptor cell death.

### Topical Eye-drop Application of Tat-µCL Protects Photoreceptors in S334ter Rats

**Figure 3:**
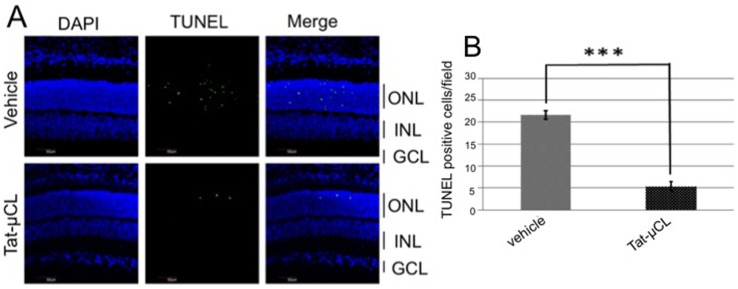
Effects of eye-drop applications of Tat-µCL on photoreceptor cell death in S334ter rats. A) TUNEL staining of retinal sections of S334ter rats treated with eye-drops containing Tat-µCL. Eye-drops containing vehicle (PBS) or 20 mM Tat-µCL were administered from PN 13 to 17 days. Eyes were enucleated at PN 18 days. Retinal sections were stained with TUNEL (green) and DAPI (blue). B) Quantitative analysis of the number of TUNEL-positive cells in the ONL at PN 18 days. Data are expressed as means ± standard deviation (n = 12 eyes (6 rats) per group). ***P<0.001 versus vehicle (*t*-test). Abbreviations: ONL, outer nuclear layer; INL, inner nuclear layer; GCL, ganglion cell layer.

We applied eye-drops containing Tat-µCL to S334ter rats, and examined the protective effects against photoreceptor degeneration (Figure 3). We placed eye-drops containing vehicle (PBS) or 20 mM Tat-µCL on the eyes of S334ter rats twice a day from PN 13 to 17 days. The eyes were enucleated on PN 18 days and the TUNEL-positive cells were detected. The number of TUNEL-positive cells in the ONL was reduced by ∼76% in Tat-µCL-treated retinas. Quantitative analysis of the number of TUNEL-positive cells showed that Tat-µCL significantly prevented photoreceptor cell death.

### Protective Effects of Tat-µCL on Retinal Function in S334ter Rats

**Figure 4:**
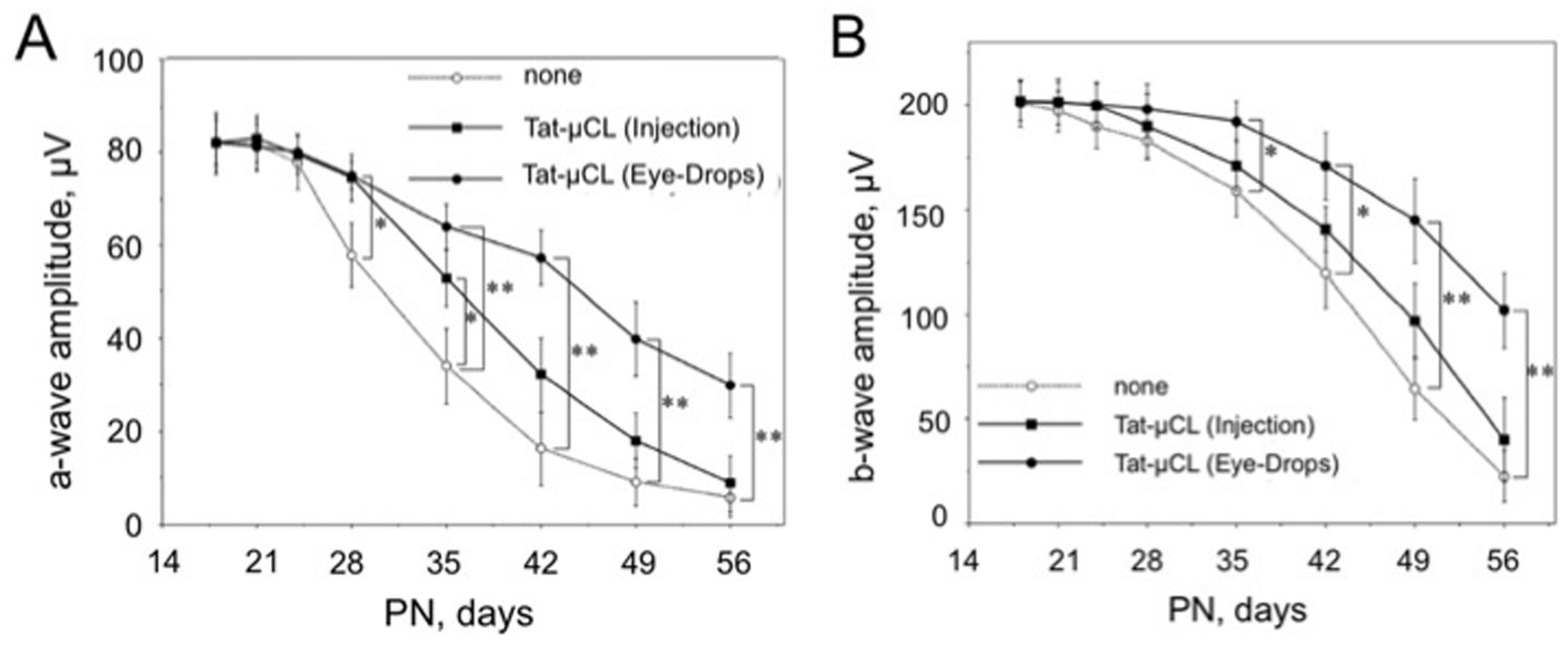
Effects of an intravitreal injection or eye drop applications of Tat-µCL on ERG in S334ter rats. S334ter rats received an intravitreal injection of 2 µl of 20 mM Tat-µCL at PN 15 days (▪). Another group of S334ter rats received eye-drops containing 20 mM Tat-µCL from PN 13 to 55 days (•). Scotopic ERGs were recorded at PN 18, 21, 24, 28, 35, 42, 49, and 56 days. A) Mean amplitudes of photoreceptor-derived a-waves. B) Mean amplitudes of Müller cells-derived b-waves. Data are expressed as means ± standard deviation (n = 8 eyes (8 rats) per group). *P<0.05 and **P<0.01 versus the none-treated group (○) (*t*-test).

Considering the protection against photoreceptor cell death with Tat-µCL, we determined the effects of Tat-µCL on preservation of retinal function in S334ter rats, using ERGs (Figure 4). We also compared the effects of intravitreal injection and eye-drop applications on ERG responses. In the group with intravitreal injection, rats received an intravitreal injection of 2 µl of 20 mM Tat-µCL at PN 15 days. In the group with eye-drop applications, we placed eye-drops containing vehicle (PBS) or 20 mM Tat-µCL on the eyes of S334ter rats twice a day from PN 13 to 56 days. Scotopic ERGs were recorded on PN 18, 21, 24, 28, 35, 42, 49 and 56 days. Although a flash ERG was performed on the dark-adapted eye, response was primarily from the rod system. Sufficiently bright flashes elicit ERGs containing an a-wave (initial negative deflection), followed by a b-wave (positive deflection). The a-wave is derived from photoreceptors, while the leading edge of the b-wave is mainly produced by the Müller cells. Tat-µCL-injected retinas showed significant preservation of a-wave at only PN 28 and 35 days (Figure 4A). However, retinas with Tat-µCL eye-drops showed significant preservation at PN 28, 35, 42, 49 and 56 days in a sustained manner. Although no significant protection of b-wave was observed in Tat-µCL-injected retinas, eye-drop application of Tat-µCL significantly and persistently preserved the b-wave at PN 35, 42, 49 and 56 days (Figure 4B).

### Topical Eye-drop Application of Tat-µCL Protects Photoreceptors in P23H Rats

**Figure 5:**
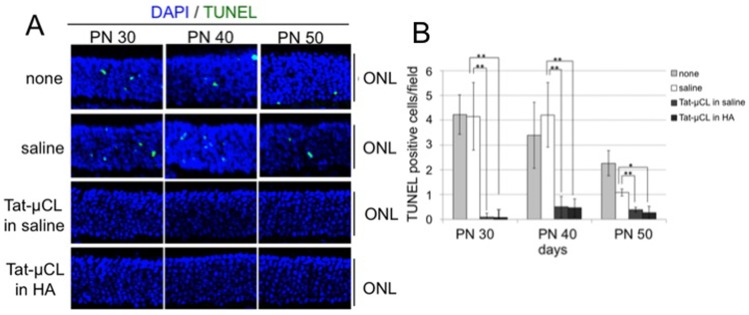
Effects of eye drop applications of Tat-µCL on photoreceptor cell death in P23H rats. A) TUNEL of retinal sections of P23H rats treated with eye-drops containing Tat-µCL. Eye-drops containing saline (PBS), 1 mM Tat-µCL in saline, or 1 mM Tat-µCL in 0.1% HA were administered from PN 14 to 49 days. Eyes were enucleated at PN 30, 40, or 50 days. Retinal sections were stained with TUNEL (green) and DAPI (blue). B) Quantitative analysis of the number of TUNEL-positive cells in the ONL at PN 30, 40, and 50 days. Data are expressed as means ± standard deviation (n = 12 eyes (6 rats) per group). *P<0.05 and **P<0.01 versus the saline-treated group (*t*-test). Abbreviations: ONL, outer nuclear layer.

**Figure 6:**
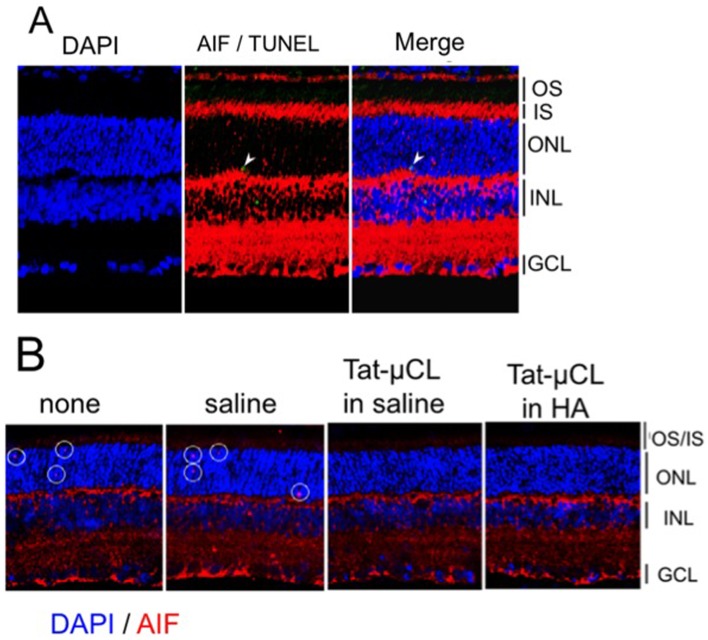
Determination of nuclear translocation of AIF in P23H rat retinas. A) Eyes were enucleated at PN 40 days, and retinal sections were stained with AIF (red), TUNEL (green) and DAPI (blue). AIF was detected in photoreceptor cell nuclei. Arrows indicate localization of AIF in TUNEL-positive photoreceptor nuclei. B) Effects of eye-drop applications of Tat-µCL on nuclear translocation of AIF in P23H rats. Eye-drops containing saline (PBS), 1 mM Tat-µCL in saline, or 1 mM Tat-µCL in 0.1% HA were administered from PN 14 to 39 days. Eyes were enucleated at PN 40 days. Retinal sections were stained with AIF (red) and DAPI (blue). White circles indicate translocation of AIF inside photoreceptor nuclei (shown by pink color). Abbreviations: OS, photoreceptor outer segment; IS, photoreceptor inner segment; ONL, outer nuclear layer; INL, inner nuclear layer; GCL, ganglion cell layer.

**Figure 7:**
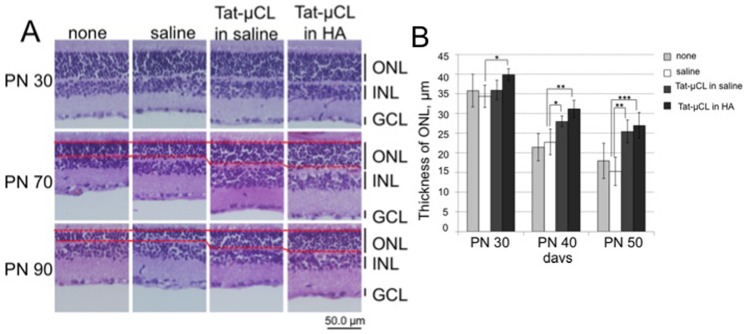
Effects of eye-drop applications of Tat-µCL on thickness of retinal layers in P23H rats. A) Eye-drops containing saline (PBS), 1 mM Tat-µCL in saline, or 1 mM Tat-µCL in 0.1% HA were administered from PN 14 to 89 days. Eyes were enucleated at PN 30, 70, or 90 days. Retinal sections were stained with hematoxylin and eosin. B) Quantitative analysis of the thickness of ONL at PN 30, 70, and 90 days. Data are expressed as means ± standard deviation (n = 12 eyes (6 rats) per group). *P<0.05, **P<0.01, and ***P<0.001 versus the saline-treated group (*t*-test). Abbreviations: ONL, outer nuclear layer; INL, inner nuclear layer; GCL, ganglion cell layer.

**Figure 8:**
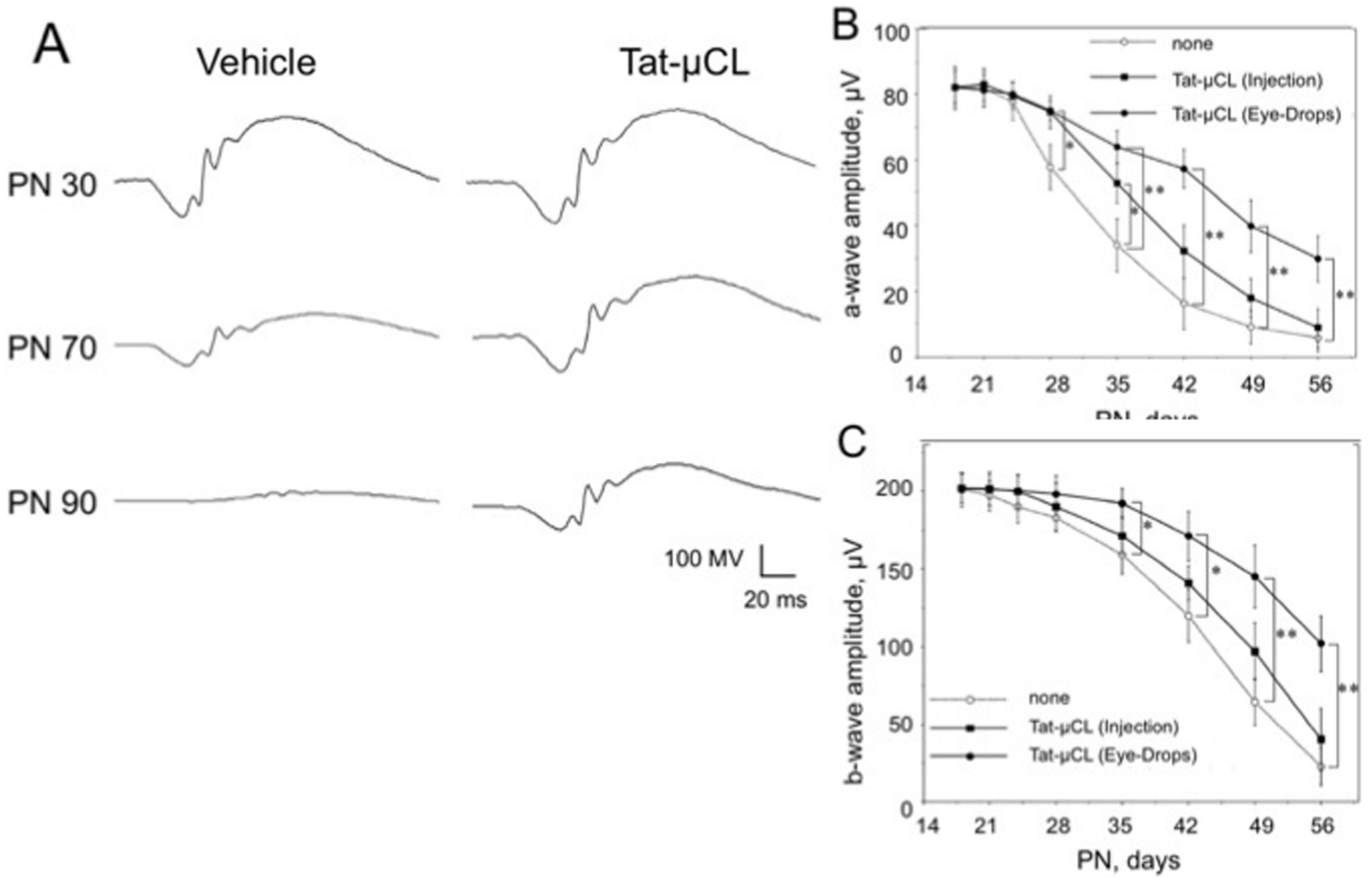
Effects of eye-drop applications of Tat-µCL on ERG in P23H rats. A) Representative ERG traces. Eye-drops containing vehicle (PBS) or 1 mM Tat-µCL in saline were administered to P23H rats from PN 14 to 89 days. Scotopic ERGs were recorded at PN 30, 70, or 90 days. B) Mean amplitudes of photoreceptor-derived a-waves. C) Mean amplitudes of Müller cells-derived b-waves. Data are expressed as means ± standard deviation (n = 8 eyes (8 rats) per group). *P<0.05 and **P<0.01 versus the vehicle-treated group (*t*-test).

Next, we examined the protective effects of Tat-µCL on another model of Rho mutants, P23H rats. Because the retinal degeneration in P23H rats progresses slowly, we examined the long-term protective effects of eye drop application of Tat-µCL against retinal degeneration in P23H rats (Figures 5–8). We used saline (PBS) or 0.1% hyaluronic acid (HA) as solvent for Tat-µCL. Eye-drops comprising PBS, 1 mM Tat-µCL in PBS, or 1 mM Tat-µCL in 0.1% HA were administered from PN 14 to 49 days. The eyes were enucleated at PN 30, 40 or 50 days, and retinal sections were stained with TUNEL. Treatment with Tat-µCL in PBS or in HA decreased TUNEL-positive cells in the ONL at PN 30, 40 and 50 days (Figure 5A). Quantitative analysis of the number of TUNEL-positive cells showed that Tat-µCL significantly prevented photoreceptor cell death (Figure 5B).

### Topical Eye-drop Application of Tat-µCL Inhibits Nuclear Trasnlocation of AIF in Photoreceptors of P23H Rats

AIF is known to be truncated by mitochondrial μ-calpain and translocate from the mitochondria to the nucleus, where it facilitates chromatin condensation and large-scale DNA fragmentation [12], [13], [15], [21]. We have previously shown that mitochondrial calpains are activated and truncate AIF, followed by the release of truncated AIF from the mitochondria into the nucleus in the initial stage of retinal degeneration in RCS rats [6]. Shinde *et al* also demonstrated a 6.5-fold increase in protein levels of truncated AIF in the cytoplasmic fraction of S334ter (line 4) compared to SD rat retinas on PN 15 days [4]. However, whether AIF is involved in photoreceptor cell death in P23H rats remains to be clarified. We therefore investigated whether AIF released from mitochondria accumulated in photoreceptor nuclei of P23H rats. We found that AIF was translocated from the mitochondria-rich inner segment to some photoreceptor nuclei in P23H rat retinas at PN 40 days (Figure 6A). In addition, one of the AIF-positive nuclei was stained with TUNEL (Figure 6A, white arrows). Western blot analyses could not detect the release of tAIF from mitochondria into cytosol (data not shown), presumably because of the small amounts. In wild-type SD rat retinas, we could not detected AIF translocation to photoreceptor nuclei [6]. Next, we determined whether eye-drop applications of Tat-µCL prevented AIF translocation to photoreceptor nuclei in P23H rats. Eye-drops containing PBS, 1 mM Tat-µCL in PBS, or 1 mM Tat-µCL in 0.1% HA were administered from PN 14 to 39 days. Eyes were enucleated at PN 40 days, and retinal sections were stained with AIF. We found that eye-drop application of Tat-µCL prevented the nuclear translocation of AIF in ONL (Figure 6B).

### Topical Eye-drop Application of Tat-µCL Prevents Thinning of the Photoreceptor Layer in P23H Rats

We determined the severity of thinning of the photoreceptor layer and the protective effects of eye-drop application of Tat-µCL in P23H rats. Eye-drops containing PBS, 1 mM Tat-µCL in saline, or 1 mM Tat-µCL in 0.1% HA were administrated from PN 14 to 89 days. Eyes were enucleated at PN 30, 70 or 90 days, and retinal sections were stained with hematoxylin and eosin. The results showed that thickness of the ONL gradually decreased from PN 30 to 90 days (Figure 7A). However, treatment with Tat-µCL clearly prevented thinning of the ONL. Quantitative analysis of ONL thickness showed that Tat-µCL significantly prevented thinning of the ONL at PN 70 and 90 days (Figure 7B).

### Topical Eye-drop Application of Tat-µCL Protects Retinal Function in P23H Rats

We examined the effects of Tat-µCL on preservation of retinal function in P23H rats using ERG (Figure 8). We placed eye-drops containing vehicle (PBS) or 1 mM Tat-µCL on the eyes of P23H rats twice a day from PN 14 to 89 days. Scotopic ERGs were recorded at PN 30, 70 and 90 days. ERG responses were seen gradually attenuate from PN 30 to 90 days (Figure 8A). However, treatments of Tat-µCL noticeably prevented attenuation of ERG response at PN 70 and 90 days. Quantitative analysis of the a- and b-wave of ERG response showed that Tat-µCL significantly prevented attenuation at PN 70 and 90 days (Figures 8B, C).

## Discussion

The present study demonstrated that mitochondrial μ-calpain inhibitory peptide, Tat-µCL, prevented photoreceptor cell death and delayed the progression of retinal degeneration in Rho transgenic S334ter and P23H rats. Although we had previously found that Tat-µCL protects against retinal degeneration in *Mertk* mutant RCS rat [7], the present results revealed that the peptide also exerted protective effects against degeneration in the most prevalent mutations in ADRP, *RHO* mutants.

Although ADRP is associated with mutations in at least 20 different genes, mutations in the Rho gene (*RHO*, OMIM 180380, accession ID U49742) are the most prevalent, identified in 30–40% of all ADRP cases [24], [25]. In addition, Rho mutations also show highly variable phenotypes depending on the location of the mutation [25]. The mutation of Rho N-terminus, the substitution of histidine for proline in the 23^rd^ amino acid (P23H), has been observed in about 12% of ADRP patients [24]. Although P23H shows a relatively mild clinical progression, C-terminal mutations such as S334ter generally exhibit a more severe clinical phenotype [25], [26]. This is probably because the C-terminal domain is important for Rho sorting to rod outer segments, and for Rho phosphorylation and binding of arrestin [31], [32]. In P23H rats, Rho appears to be mis-folded in the ER [33], [34]. In S334ter rats, Rho is truncated at the C-terminus and lacks the last 15 amino acid residues and is thus mis-localized in the cytoplasm or cell membrane of photoreceptors through interference with post-Golgi trafficking [32], [35], [36]. In any case, as a result of the inhibition of both sorting and dysfunction of Rho, photoreceptor physiology is changed and cell death results [2], [36]–[41].

The two different mutants show roughly comparable mechanisms of cell death mediated by calpains, AIF, ER stress, PARP, or caspase-3 [3]–[5]. Among these causative factors, the present study inhibited the mitochondrial μ-calpain and AIF pathway using the Tat-µCL, because several studies have demonstrated that the activation of calpains and translocation of AIF from mitochondria occurred in the initial stage of photoreceptor cell death in S334ter and P23H rats [3], [4]. Our results show that intravitreal injection or eye-drop application of the Tat-µCL inhibited photoreceptor cell death in the early stages of degeneration in S334ter and P23H rats (Figures 2, 3 and 5). In P23H rats, the peptide also prevented nuclear translocation of AIF in the photoreceptor (Figure 6B). These protections would have beneficial effects in delaying the progression of visual disturbance and thinning of the photoreceptor layer (Figures 4, 7 and 8). Our results suggest that retinal degeneration occurs via the mitochondrial μ-calpain and AIF-dependent pathway in not only RCS rats, but also Rho transgenic S334ter and P23H rats. In addition, inhibition of this pathway would delay retinal degeneration in RP resulting from Rho gene mutations. The present results also suggest that inhibition of photoreceptor cell death by the Tat-µCL may be mutation-independent.

In contrast, we could not completely prevent the attenuation of ERG responses and thinning of the photoreceptor layer. In addition to inhibition of the mitochondrial μ-calpain and AIF pathway, we should consider ER stress, oxidative stress, and caspase activation induced in the middle to late stages of retinal degeneration in S334ter and P23H rats. In particular, we should also take the mis-folding and mis-sorting of Rho protein into account. Recent studies have revealed that ER stress response or unfolded protein response (UPR) is involved in retinal degeneration in mouse, rat, and Drosophila models of RP [4], [5], [42]–[45].

In addition to RP, the inhibition of calpain activity seems likely to be beneficial for protection against the retinal ganglion cell (RGC) death seen in glaucoma. Recent studies have shown that calpains are activated and inhibition of calpain activity attenuates RGC death in rat models of glaucoma [46]–[48]. Maintenance of RGC mitochondria is also key to neuroprotection in glaucoma [49]. Although it remains to be elucidated whether RGC degeneration involves the mitochondrial μ-calpain and AIF-dependent pathway, the intracellular Ca^2+^ elevation in RGC [50] could trigger the activation of mitochondrial calpains as well as cytosolic calpains, and activated mitochondrial calpains would contribute to RGC death via AIF truncation/activation in glaucoma. We thus believe that Tat-µCL has therapeutic potential for preventing RGC degeneration in glaucoma. Very recently, Das *et al* demonstrated that calpain inhibition could prevent inflammation, apoptosis, and axonal degeneration in a rat model of acute optic neuritis, experimental autoimmune encephalomyelitis [51]. The Tat-µCL may also offer widespread effects in treating optic neuritis.

In summary, the mitochondrial μ-calpain and AIF pathway is involved in the early stage of retinal degeneration in Rho transgenic S334ter and P23H rats, and inhibition of this pathway using Tat-µCL leads to effective treatment of RP involving Rho mutation.
