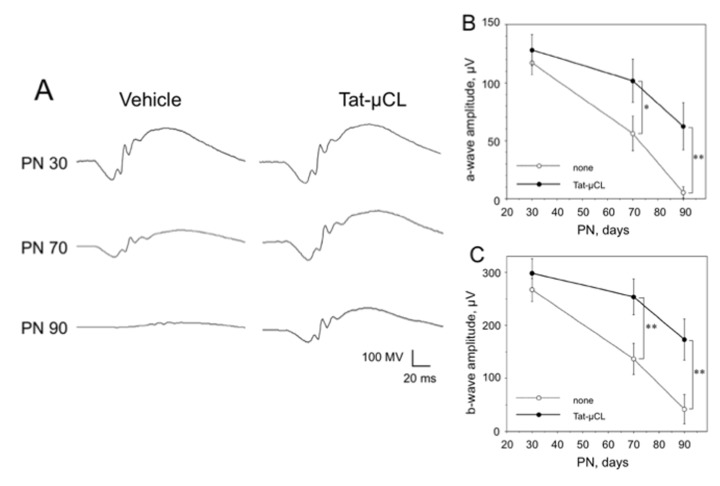# Correction: Inhibitory Peptide of Mitochondrial μ-Calpain Protects against Photoreceptor Degeneration in Rhodopsin Transgenic S334ter and P23H Rats

**DOI:** 10.1371/annotation/7a8aaf1d-e968-4b39-abb0-867d6078b2af

**Published:** 2013-09-10

**Authors:** Taku Ozaki, Sei-ichi Ishiguro, Satoshi Hirano, Ayaka Baba, Tetsuro Yamashita, Hiroshi Tomita, Mitsuru Nakazawa

The published Figure 8 is incorrect. Please view corrected Figure 8 here: 

**Figure:**